# Resting-state EEG and machine learning to investigate cortical connectivity as a biomarker in chronic mTBI

**DOI:** 10.3389/fneur.2025.1721726

**Published:** 2026-01-26

**Authors:** William J. Marshall, Amy N. Conner, Alexandra P. Key, Tonia S. Rex

**Affiliations:** 1 Vanderbilt Eye Institute, Vanderbilt University Medical Center, Nashville, TN, United States; 2 Neuroscience Graduate Program, Vanderbilt University, Nashville, TN, United States; 3 Department of Pediatrics, Emory University School of Medicine, Atlanta, GA, United States; 4 Department of Hearing and Speech Sciences, Vanderbilt University Medical Center, Nashville, TN, United States

**Keywords:** mild TBI, resting-state EEG, multivariate interaction measure, functional connectivity, machine learning, classification, biomarker

## Abstract

**Introduction:**

Mild traumatic brain injury (mTBI) is a heterogeneous condition with long-term sequelae, yet diagnosis in the chronic stage remains limited by reliance on acute criteria and subjective reports. Objective biomarkers are needed, as current blood-based markers show diagnostic value primarily in the acute and subacute phases. Resting-state EEG (RS-EEG) can capture large-scale network disruptions through functional connectivity (FC) and microstate analysis, but its role in chronic mTBI is unclear.

**Methods:**

We tested whether RS-EEG features distinguish chronic mTBI from controls and predict symptom burden. This observational case–control study included 44 participants (18 chronic mTBI, 26 controls). Source-reconstructed EEG was analyzed for spectral power, microstate metrics, and FC using the Multivariate Interaction Measure (MIM). Elastic Net and XGBoost models classified injury status and predicted symptom severity, with feature robustness evaluated across full and reduced electrode montages.

**Results:**

Participants with mTBI showed no group differences in spectral power or microstate metrics but demonstrated significantly elevated FC across theta, beta, gamma, and broadband frequencies. Connectivity increases were stable across reduced montages and persisted up to 8 years post-injury. Classification models using MIM achieved AUCs of 0.79–0.89 for injury status and 0.82–0.87 for symptom severity, outperforming demographic models. Resting-state EEG FC provides a sensitive biomarker of chronic mTBI, distinguishing cases from controls and correlating with symptom severity.

**Discussion:**

The persistence of network alterations years after injury suggests lasting changes in brain activity associated with chronic symptom burden. These findings support the use of RS-EEG–derived FC as a noninvasive and scalable biomarker of chronic mTBI.

## Introduction

Mild traumatic brain injury (mTBI) is a heterogeneous neurological condition with potential for persistent symptoms and neurophysiological disruption (1). Yet, diagnosis in the chronic stage remains challenging. Definitions of “chronic” mTBI vary across studies, with proposed thresholds ranging from ≥3 to ≥12 months post-injury (2–5). At this stage, acute measures such as the Glasgow Coma Scale (GCS) are not administered, and neuroimaging is often not obtained, leaving clinicians to rely on symptom reports that may not reflect underlying brain dysfunction. Up to 35–53% of individuals remain symptomatic more than 5–10 years post-injury (4, 6), emphasizing the need for objective biomarkers to detect and monitor chronic impairment.

The recently proposed clinical-biomarker-imaging modifiers (CBI-M) framework highlights the importance of functional biomarkers to complement symptom-based criteria to enhance classification accuracy (7). While blood-based biomarkers such as glial fibrillary acidic protein (GFAP) and ubiquitin C-terminal hydrolase L1 (UCH-L1) show diagnostic utility in the acute (within 24 h) and subacute (within 3 months) phases of mTBI (8), their role in identifying or monitoring long-term dysfunction (>6 months–1 year) remains underexplored. Neurophysiological measures, particularly resting-state EEG (RS-EEG), offer a scalable and noninvasive alternative for probing persistent network dysfunction.

RS-EEG provides a window into large-scale brain dynamics. Functional connectivity (FC) analyses quantify the efficiency of communication between brain regions, while microstate analysis characterizes rapid transitions between global brain states (9, 10). Both FC and microstates show alterations across neurological and psychiatric disorders, with changes manifesting as either increases or decreases depending on the condition (11–15). However, their utility in chronic mTBI, particularly in individuals with diverse injury mechanisms and extended post-injury durations, remains unclear. mTBI is known to disrupt distributed cortical hubs involved in attention, sensory integration, and executive function (16), and our prior work in this cohort identified atypical auditory and visual evoked responses years after injury (17, 18), suggesting that sensory processing deficits may reflect broader network dysfunction.

To address this gap, we hypothesized that individuals with chronic mTBI would show altered RS-EEG connectivity and microstate dynamics, reflecting persistent network disruption. Because scalp EEG signals can be spatially ambiguous, we applied source reconstruction to enhance anatomical specificity, enabling biologically interpretable network-level analyses. We further tested whether these features could classify injury status, predict symptom burden, and remain robust under reduced electrode montages, supporting their potential as clinically scalable biomarkers.

## Materials and methods

### Participants

This observational case–control diagnostic study enrolled adults aged 18–72 with either a history of mild traumatic brain injury (mTBI; GCS 13–15) or no history of head injury (controls). Exclusion criteria included severe TBI (GCS < 13), eye or ear disease (e.g., Meniere’s disease, glaucoma), metallic implants incompatible with neuroimaging, pregnancy, or age <18. All participants had normal hearing thresholds and 20/20 best-corrected visual acuity. Corrective lenses were permitted.

**Figure 1:**
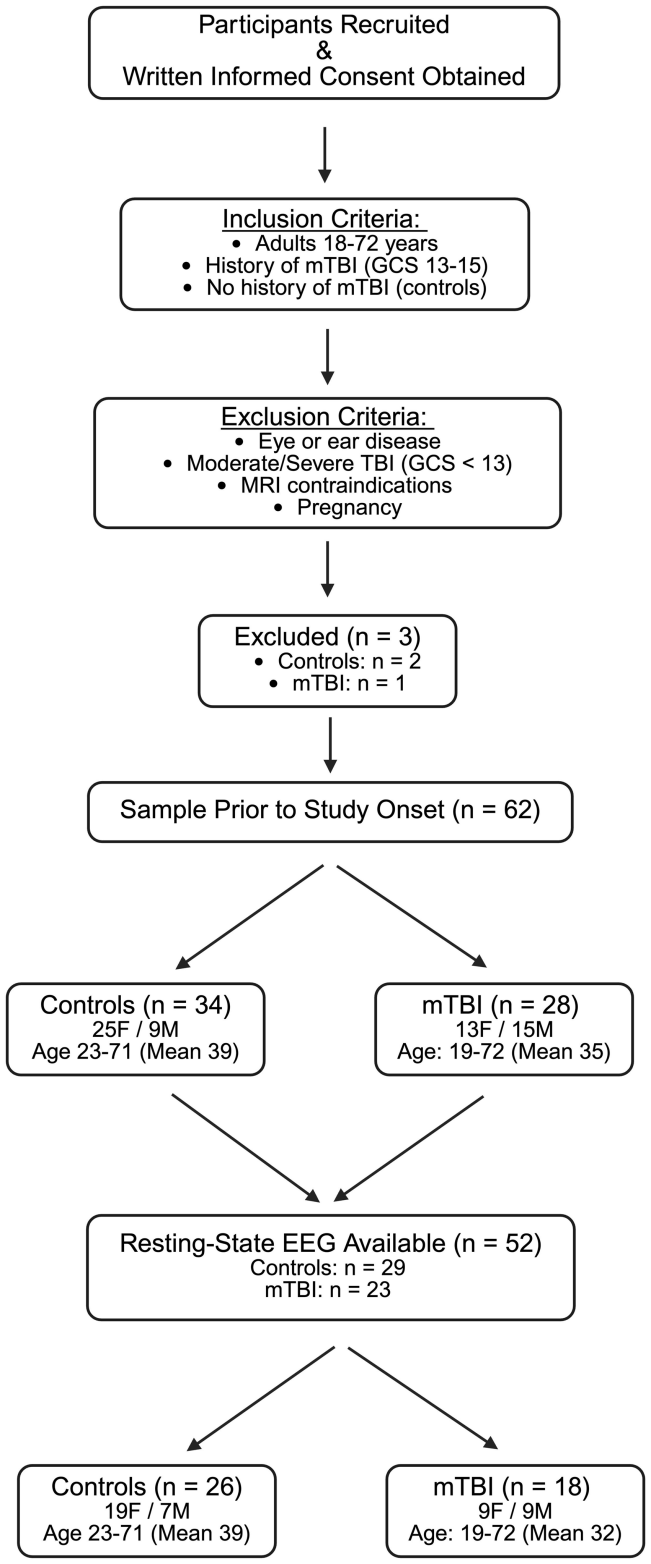
Participant recruitment and study flowchart. Adult participants (≥18 years) provided written informed consent before any study procedures. Individuals were eligible if they had a history of mild traumatic brain injury (mTBI; Glasgow Coma Scale [GCS] 13–15) or no history of mTBI (controls). Exclusion criteria included eye or ear disease, moderate/severe TBI (GCS < 13), contraindications to MRI, age <18 years, or pregnancy. Three participants were excluded before study completion. The final sample included 62 participants (28 mTBI, 34 controls), with resting-state EEG data available for a subset (23 mTBI, 29 controls) due to attrition in the multi-day study design.

A total of 65 individuals were recruited, with 28 participants with mTBI (mean age 35, SD = 13.08, range = 19–72, 13 female) and 34 controls (mean age 39, SD = 11.34, range = 23–71, 25 female) included after exclusions (Figure 1). Out of 62 included participants, resting-state EEGs were completed for 23 participants with mTBI and 29 controls (Figure 1). mTBI history was confirmed via medical records or structured history, including injury mechanism and treatment. Mechanisms included motor vehicle accidents (*n* = 9), falls (*n* = 7), non-violent blunt trauma (*n* = 3), and sports injuries (*n* = 4). The average time since last mTBI was 7.25 ± 6.71 years.

The study was approved by the Vanderbilt University Medical Center Institutional Review Board (IRB #171061). Written informed consent was obtained from all participants before any study procedures, including screening for inclusion and exclusion criteria, data collection, or analysis. All participants voluntarily participated and were compensated for their time in accordance with IRB guidelines.

### Procedure

Participants completed the Neurobehavioral Symptom Inventory (NSI), a validated 22-item self-report measure of post-concussive symptoms (19). Resting-state EEG (RS-EEG) was recorded in a quiet, sound-treated room. Data were collected with a 128-channel hydrocel net (Electrical Geodesics, Inc., Eugene, OR) at 1,000 Hz, referenced to Cz. Electrode impedances were kept <50 kΩ. Recordings included 3 min eyes-open (EO) followed by 3 min eyes-closed (EC). The fixed order minimized drowsiness and artifact variability. During EO, participants fixated on a central cross to reduce eye movements. Condition transitions were signaled by a 500 Hz auditory beep, with event markers inserted into the EEG file for alignment. This was part of a larger study, components of which have been published elsewhere (17, 18).

### EEG preprocessing and source reconstruction

#### Preprocessing

Data were preprocessed in MATLAB (MathWorks, Natick, MA) using EEGLAB (20) and a customized version of the Maryland Analysis of Developmental EEG (MADE) pipeline (21). As part of the MADE pipeline, signals were bandpass filtered at 0.1–50 Hz using the FIRfilt plugin (22), then segmented into 2,000-ms epochs. Bad channels were identified and interpolated, and epochs with >10% interpolated electrodes or >±100 μV amplitude were rejected. The Fully Automated Statistical Thresholding for EEG artifact Rejection (FASTER) algorithm aided channel-level artifact detection (23), and artifact-related independent components were identified using ADJUST (24). Eyes-closed recordings were used for primary analyses due to lower artifact susceptibility (25).

#### Source reconstruction

Source reconstruction was performed using ROIConnect (EEGLAB plugin) with the Montreal Neurological Institute (MNI) Boundary Element Model and the Colin27 template (26–28). Cortical activity was estimated via exact low-resolution electromagnetic tomography (eLORETA) and parcellated into Desikan-Killiany atlas regions to extract biologically interpretable ROI time series (29, 30). All subsequent analyses of spectral power, microstates, and functional connectivity were conducted using these source-reconstructed ROI time series.

### EEG analyses

#### Power spectra analysis

Power spectral density (PSD) was computed to assess frequency-specific neural oscillations, given prior reports of spectral alterations in neurotrauma (31, 32). Data were analyzed in EEGLAB with 20,000 permutations and False Discovery Rate (FDR) correction (*p* < 0.05) (33). The FOOOF toolbox was used to separate narrowband oscillations from the aperiodic 1/f component (34). Power was examined across delta (1–4 Hz), theta (4–8 Hz), alpha (8–12 Hz), beta (13–30 Hz), and gamma (30–50 Hz) bands.

#### Microstate analysis

Microstate analysis across 1–50 Hz was conducted in MicrostateLab (EEGLAB plugin) (35). Global Field Power peaks were clustered (*k* = 4–7) using k-means, with optimal solutions selected via meta-criteria in Cartool (36). Group-averaged maps were matched to canonical templates based on the highest correlation, and temporal dynamics (duration, coverage, occurrence) were derived by back-fitting. Group differences were assessed using Topographic Analysis of Variance (TANOVA) in RAGU with 100,000 permutations and FDR correction (37).

#### Functional connectivity (MIM)

Functional connectivity was computed using the Multivariate Interaction Measure (MIM), which reduces the impact of volume conduction by focusing on non-instantaneous interactions in the frequency domain. Connectivity was estimated for the broadband range (1–50 Hz) and standard frequency bands (delta [1–4 Hz], theta [4–8 Hz], alpha [8–12 Hz], beta [13–30 Hz], gamma [30–50 Hz]). Outliers were capped at 3 × IQR to reduce undue influence. Group differences were tested with type III ANCOVA controlling for age and sex. Because Levene’s test indicated heteroscedasticity, robust standard errors were applied (38). Effect sizes were estimated with bootstrapped partial *η*^2^ and 95% confidence intervals.

#### Reduced ROI analysis

**Figure 2:**
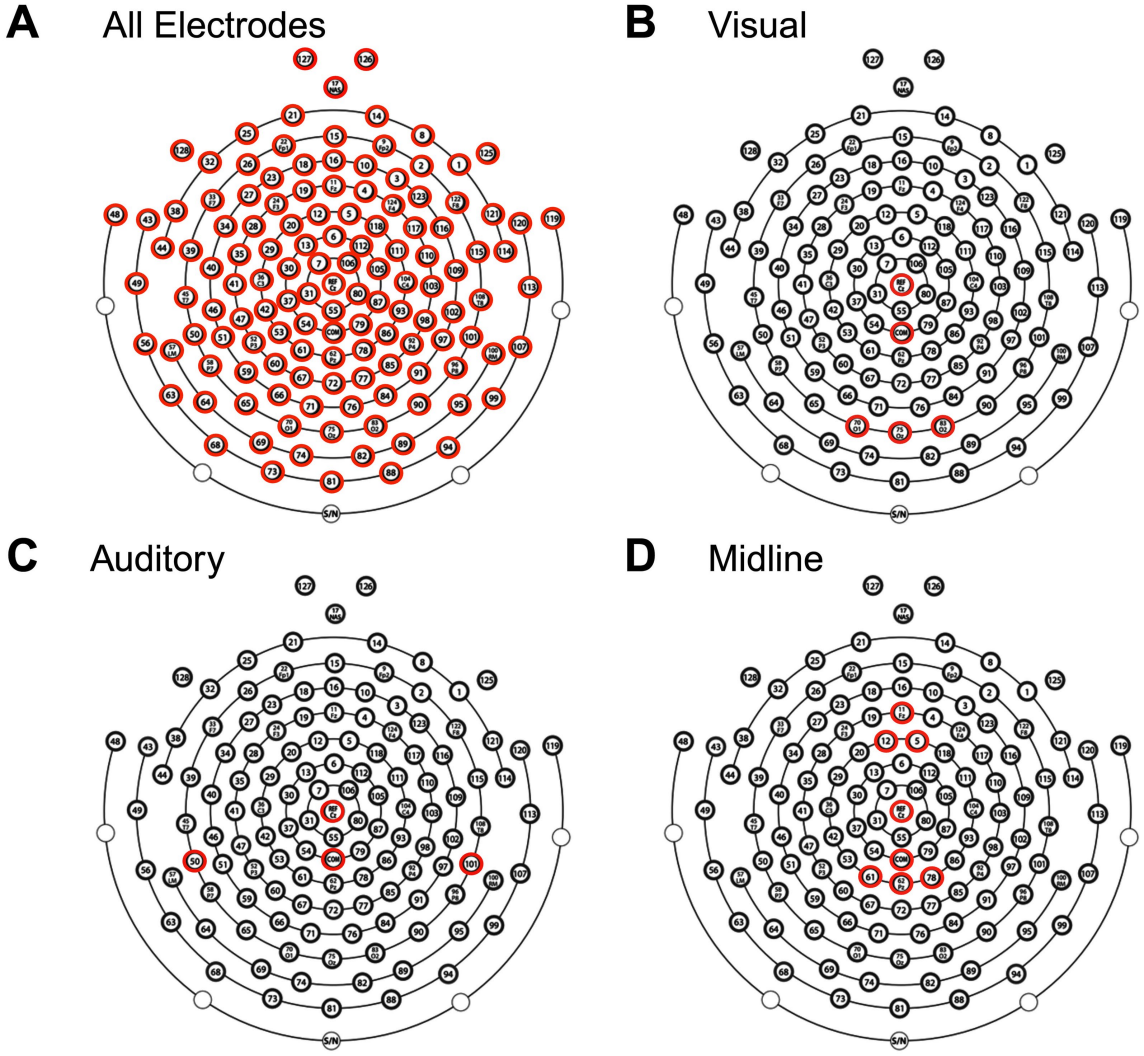
Electrode selection for EEG analysis. Topographical layout of the EEG cap showing electrodes included in four analyses: **(A)** All electrodes, **(B)** visual subset, **(C)** auditory subset, and **(D)** midline subset. Highlighted electrodes (red) indicate those used in each respective analysis, including the reference (Cz) and common ground electrode (COM).

To evaluate clinical scalability, we repeated FC analyses using reduced ROI subsets designed to approximate sparse EEG montages. These subsets reflect electrode coverage commonly used in clinical visual and auditory recordings but were applied here to resting-state EEG. No evoked potentials were recorded. The selected regions prioritize accessible electrode locations and target sensory systems that have previously been shown to exhibit deficits in mTBI (17, 18). The reduced sets included (1) occipital (visual), (2) superior temporal (auditory), and (3) frontoparietal regions (Figure 2). Results were FDR-corrected across frequency bands and montage conditions.

#### Classifier analysis

To evaluate whether FC features distinguished chronic mTBI from controls, we applied three complementary classification methods: (1) threshold-based cutoffs, which use a single optimal decision boundary (Youden’s index) to maximize sensitivity and specificity (39); (2) Elastic Net logistic regression, a linear model that combines L1/L2 regularization to handle correlated predictors and reduce overfitting (40); and (3) XGBoost gradient-boosted decision trees, a nonlinear ensemble method capable of modeling higher-order feature interactions (41). This combination allowed comparison of simple interpretable models versus more flexible machine learning approaches. MIM values were z-scored for Elastic Net but left unscaled for XGBoost. Hyperparameters were tuned with 5-fold cross-validation. Classification performance was quantified with ROC receiver operating characteristic (ROC) curves and area under the curve (AUC, 95% CIs from 20,000 bootstraps). Model significance was assessed with 20,000 permutation tests of shuffled labels. Models were implemented in MATLAB (Elastic Net) and Python (XGBoost with scikit-learn and xgboost).

#### Symptom severity prediction analysis

Associations between FC and post-concussive symptoms were examined using NSI total score. Linear regression tested whether MIM values predicted total NSI scores (controlling for age and sex). For secondary analyses, participants were dichotomized into no-mild (NSI total score <25) vs. moderate–severe (NSI total score ≥25) symptom groups, and classification was performed using Elastic Net and XGBoost. Models were tuned with 5-fold cross-validation, and performance was quantified with ROC AUCs (95% CIs via 20,000 bootstraps). Statistical significance was assessed with 20,000 label-permutation tests.

## Results

After exclusions for incomplete recordings (*n* = 5, 2 controls) and excessive artifacts (*n* = 3, 1 control), 44 participants were included in the final analysis. The control group (*n* = 26) had a mean age of 39.6 years (SD = 11.5) and was 73% female, while the mild TBI group (*n* = 18) had a mean age of 32.0 years (SD = 11.4) and was 50% female. Age distributions were compared between included and excluded participants. For mTBI participants, Shapiro–Wilk indicated non-normality in the included group (*p* = 0.003), so a Mann–Whitney *U* test was used. Excluded cases were significantly older (*U* = 53.0, *p* = 0.041). For controls, both groups were approximately normal (*p* > 0.07), and Welch’s *t*-test showed no difference (*t* = 0.88, *p* = 0.39). Thus, the final analysis sample contained younger mTBI participants relative to excluded cases, while control demographics were preserved. To account for this potential confound, age was included as a covariate in all analyses.

Among mTBI participants, the average time since their last mTBI was 7.7 years (SD = 7.0), consistent with chronic-phase recovery. Injury mechanisms are detailed in Table 1. There were no significant differences in injury time or mechanism between excluded and included participants with mTBI (*p* > 0.05).

**Table 1:** Participant demographics.

Variable	Control (*n* = 26)	mTBI (*n* = 18)
Age, mean (SD)	39.6 (11.5)	32.0 (11.4)
Sex, % Female	73%	50%
# of mTBIs, *n* (%)		
1	–	9 (50%)
2	–	1 (6%)
3	–	6 (33%)
4	–	2 (11%)
Did not report	–	0 (0%)
Years since last mTBI, *n* (%)		
<1	–	1 (6%)
1–2.9	–	6 (33%)
3–6.9	–	2 (11%)
7–9.9	–	2 (11%)
>10	–	6 (33%)
Did not report	–	1 (6%)

mTBI, mild traumatic brain injury.

After preprocessing, average data retention was lower in cases than controls (mTBI: mean = 0.68, SD = 0.32; Control: mean = 0.92, SD = 0.12; Mann–Whitney *U* test, *p* = 0.009). For eyes-open EEG, retention did not differ between groups (mTBI: mean = 0.78, SD = 0.28; Control: mean = 0.86, SD = 0.17; Mann–Whitney *U* test, *p* = 0.424). Retention was lower in mTBI participants during eyes-closed recordings, likely reflecting increased artifact, but all participants retained adequate data for valid analysis. Moreover, the consistency of eyes-open retention across groups suggests that the observed group differences are not attributable to general data quality disparities.

### Resting-state spectral power is preserved after chronic mTBI

**Figure 3:**
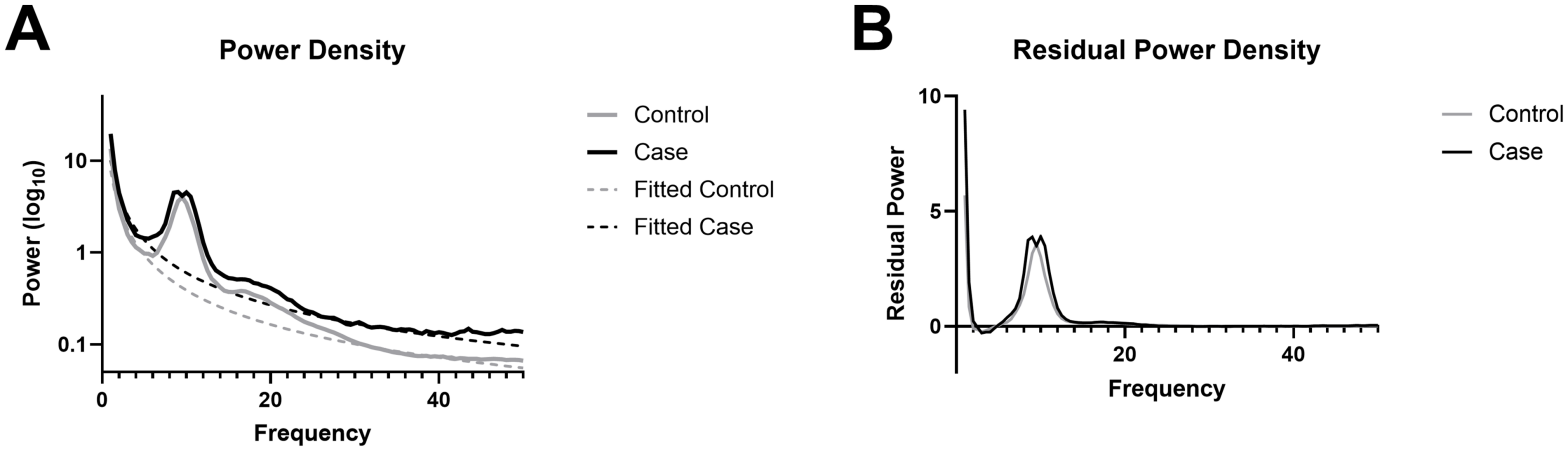
Power spectral density. **(A)** Power spectra with an aperiodic fit overlay. **(B)** Periodic residual from the removal of the aperiodic fit from raw power spectra.

No significant group differences were observed in eyes-closed resting-state power spectra across the full 0–50 Hz range (*p* > 0.05; Figure 3A), indicating that overall spectral content remained comparable between mTBI and control participants. This pattern held after removing the aperiodic background using FOOOF, isolating true oscillatory components. Group comparisons of the periodic spectra also revealed no significant differences (*p* > 0.05; Figure 3B), suggesting that mTBI does not produce broad alterations in canonical frequency bands during rest.

### Microstate topographies and temporal dynamics are unaltered after chronic mTBI

**Figure 4:**
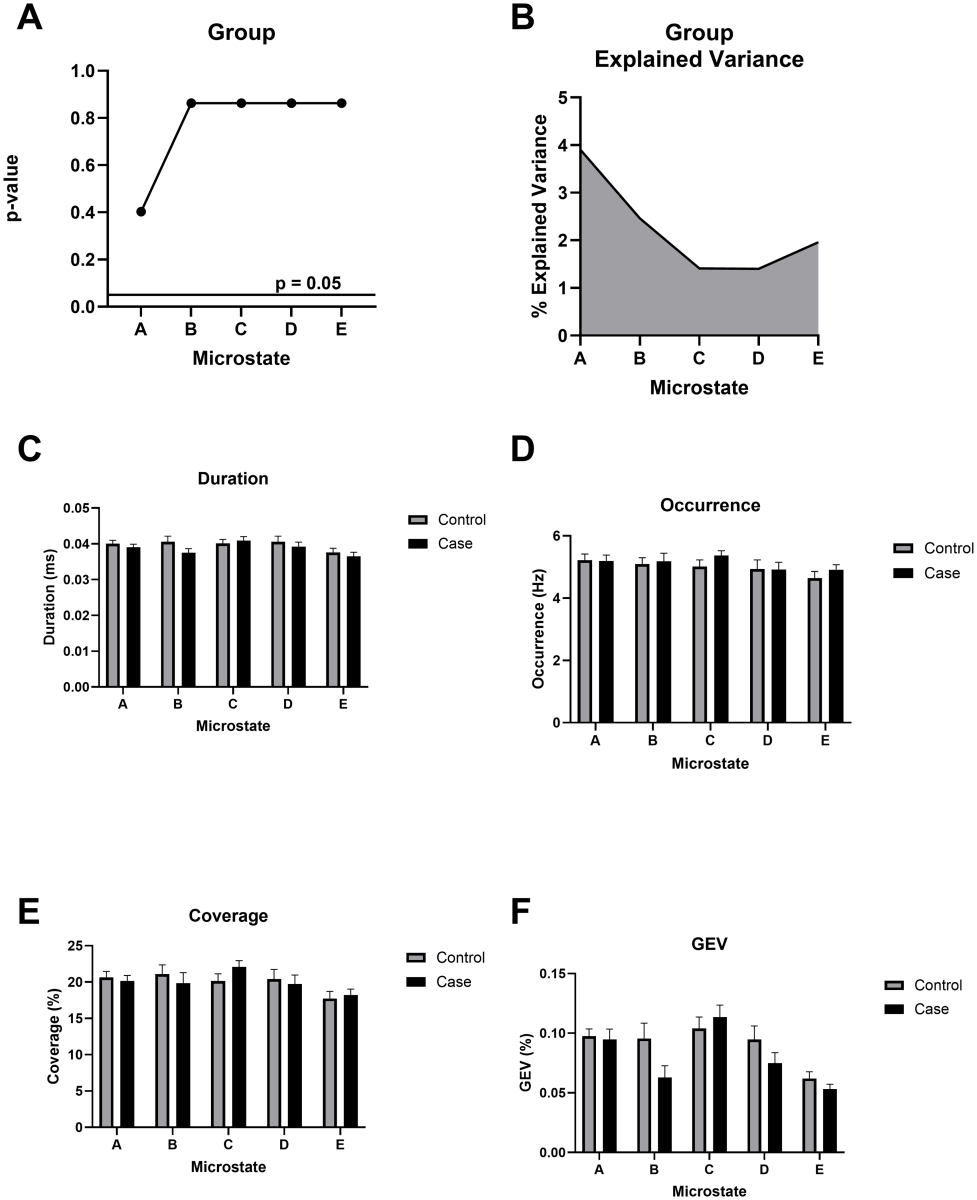
Microstate topographical analysis of variance (TANOVA). **(A)** *p*-value of the topographical difference based on group (mild TBI/control) across all microstates. All *p*-values use FDR correction. **(B)** Cumulative explained variance (CEV) based on group. **(C)** Duration (in ms) reflects the average length of each microstate. **(D)** Occurrence (in Hz) represents the frequency of each microstate per second. **(E)** Coverage denotes the proportion of total time occupied by each microstate. **(F)** Global explained variance (GEV) quantifies the proportion of EEG variance explained by each microstate. No significant group differences were observed across any microstate metric **(A–F)**.

Microstate topographies, reflecting canonical spatial configurations of scalp potential fields, were statistically indistinguishable between the mTBI and control groups across 5 optimal microstates, as assessed using TANOVA (Figures 4A,B). This indicates that the overall spatial organization of microstates was preserved in individuals with chronic mTBI. Further, the temporal dynamics of the backfitted microstate sequences, including duration, occurrence, coverage, global explained variance, and transition probabilities, also showed no significant group differences (Figures 4C–F), suggesting preserved microstate temporal dynamics in individuals with chronic mTBI.

### Chronic mTBI is associated with widespread cortical hyperconnectivity

**Figure 5:**
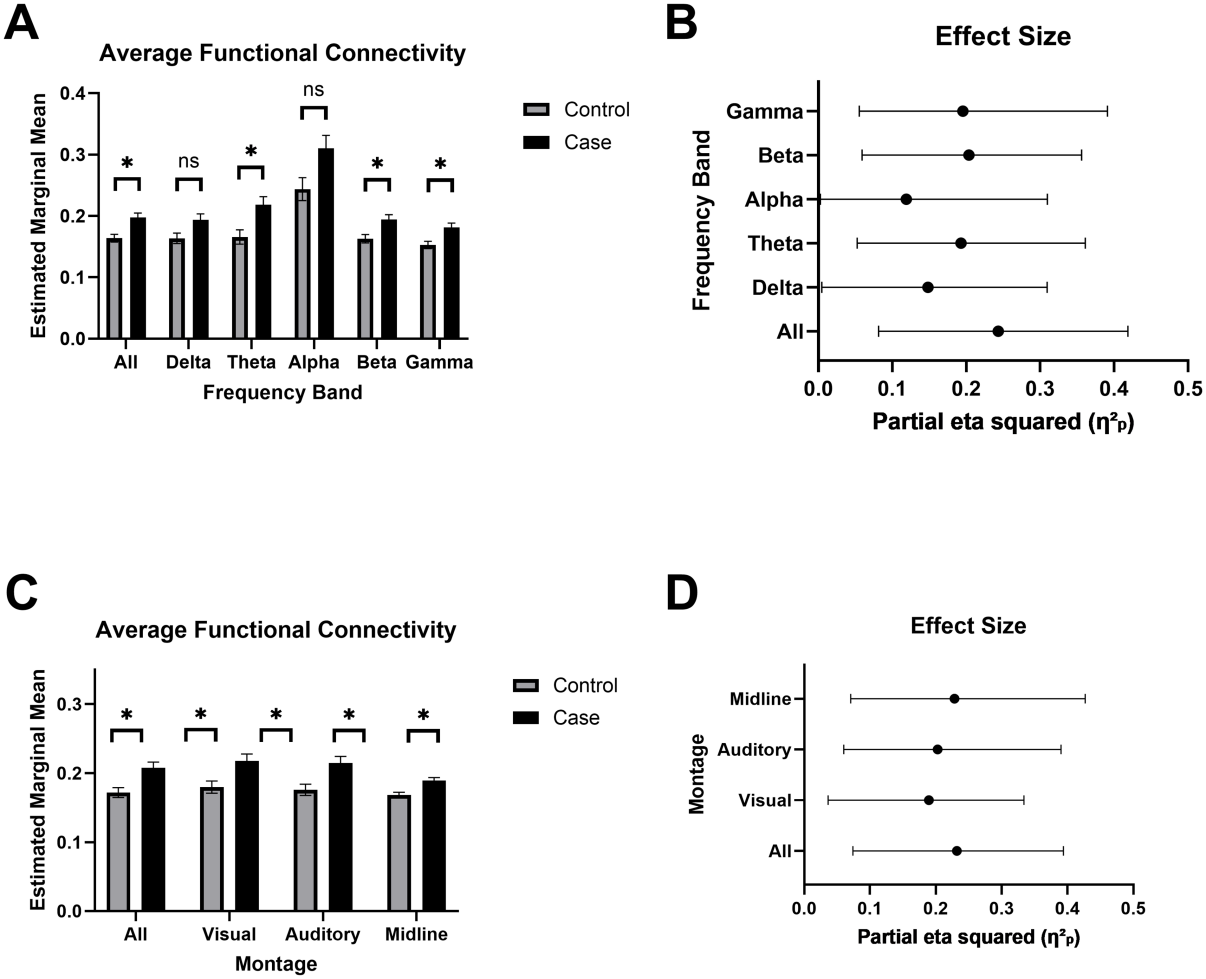
Increase in estimated functional connectivity. **(A)** Estimated marginal mean MIM (multivariate interaction measure) across frequency bands: delta (1–4 Hz), theta (4–7 Hz), alpha (8–12 Hz), beta (13–30 Hz), and gamma (30–45 Hz). Bars represent mean ± SEM for each group. Asterisks indicate FDR-corrected statistical significance between groups (*p* < 0.05 = *, *p* < 0.01 = **, *p* <  0.001 = ***). **(B)** Effect sizes for each frequency band are reported as partial eta squared (*η*^2^_*p*_), indicating the proportion of variance accounted for by MIM. Bars represent mean ± SEM for each group. **(C)** Estimated marginal mean MIM across subregions to simulate alternative EEG montages. **(D)** Effect sizes for each montage are reported as partial eta squared (*η*^2^_*p*_), indicating the proportion of variance accounted for by MIM.

The mild TBI group exhibited significantly higher functional connectivity compared to controls across all frequencies and within the theta, beta, and gamma bands (*p* < 0.05), with no group differences in the delta or alpha bands (Figure 5A). In frequency bands showing significant effects, group membership (i.e., mTBI or control) accounted for an average of 21% of the variance (Figure 5B). These group differences remained robust when using reduced ROI subsets approximating clinically relevant EEG configurations, including occipital (visual), superior temporal (auditory), and midline-only montages (Figure 5C). Across these subsets, group explained an average of 23% of the variance in connectivity, again reflecting increased connectivity in the mTBI group relative to controls (Figure 5D).

### Machine learning captures robust differences in functional connectivity after chronic mTBI

**Figure 6:**
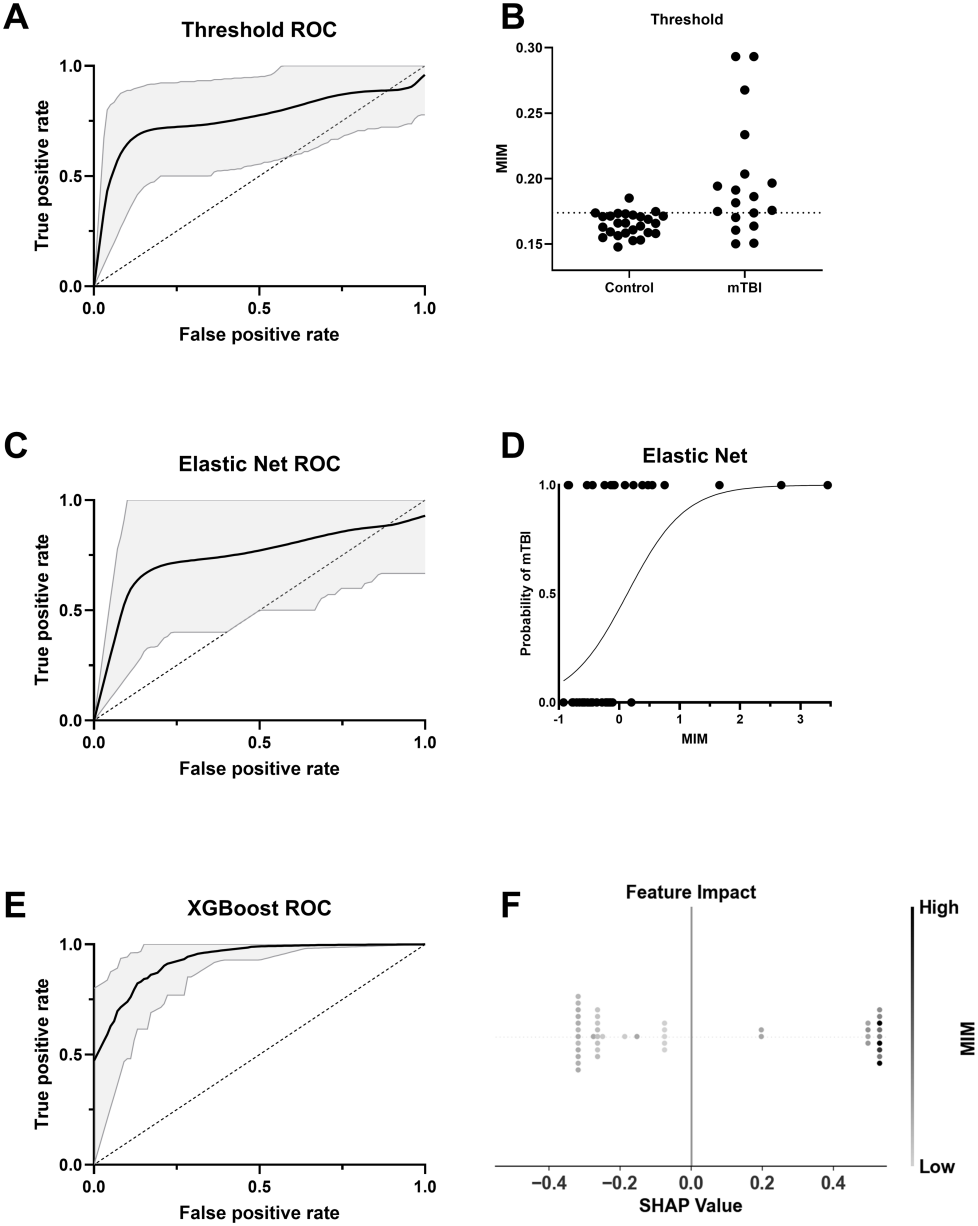
Classifier performance and interpretability for group prediction based on MIM. **(A)** ROC curve for a threshold-based classifier using the optimal Youden’s J statistic from *z*-scored MIM. The black line indicates the mean ROC curve across 20,000 bootstraps, with 95% confidence interval shaded in gray. **(B)** Distribution of normalized MIM values across Control and mTBI groups. The dashed line indicates the optimal threshold used in A. **(C)** ROC curve for an Elastic Net classifier using MIM as the predictor. The solid line shows the average ROC across bootstraps, with 95% CI shaded. **(D)** Probability curve for Elastic Net classifier output as a function of MIM. The logistic decision function exhibits a higher probability of mTBI with increasing MIM. **(E)** ROC curve for an XGBoost classifier using MIM as the predictor. **(F)** SHAP summary plot from the XGBoost model. Each dot represents an individual’s SHAP value, indicating feature impact on model output, with color based on the strength of *z*-scored MIM.

We evaluated the utility of functional connectivity (MIM) for classifying chronic mTBI from control participants using three classification approaches: a threshold-based classifier, Elastic Net regression, and XGBoost. Using the raw MIM values, the optimal threshold (0.174) yielded an AUC of 0.793 (95% CI: 0.615–0.940) (Figure 6A). The Elastic Net model (*α* = 0.03) produced a similar AUC of 0.790 (95% CI: 0.524–1.000) (Figure 6C). Both the threshold-based and Elastic Net models are acceptable and close to the 0.8 threshold commonly interpreted as excellent classification performance (42) (Figures 6B, D). The XGBoost model achieved the highest accuracy with an AUC of 0.887 (95% CI: 0.785–0.943) (Figure 6E). SHAP analysis indicated that higher MIM values were strongly associated with increased likelihood of mTBI classification (Figure 6F), reinforcing the discriminative power of functional connectivity, even in more complex models. All permutation tests yielded *p*-values less than 0.05, confirming that the observed AUCs were unlikely to occur by chance (Supplementary Figures S2A–C).

Model performance varied when age and sex covariates were included. Elastic Net performance decreased slightly, with an AUC of 0.765 (95% CI: 0.517–0.967) (Supplementary Figure S3A), possibly due to overfitting or noise introduced by additional predictors in the context of a modest sample size. In contrast, XGBoost performance improved, reaching an AUC of 0.901 (95% CI: 0.844–0.944) (Supplementary Figure S3C), suggesting better accommodation of additional covariates. Permutation tests again confirmed model significance (*p* < 0.05) (Supplementary Figures S3B,D). When MIM was removed from the models, performance declined substantially: the Elastic Net dropped to an AUC of 0.641 (95% CI: 0.438–0.867) and XGBoost to 0.835 (95% CI: 0.701–0.912; Supplementary Figures S4A,C). Despite this, models remained statistically significant (*p* < 0.05). Collectively, these results highlight MIM’s strong predictive contribution across models.

### Cortical hyperconnectivity after chronic mTBI reflects neurobehavioral symptom severity

**Figure 7:**
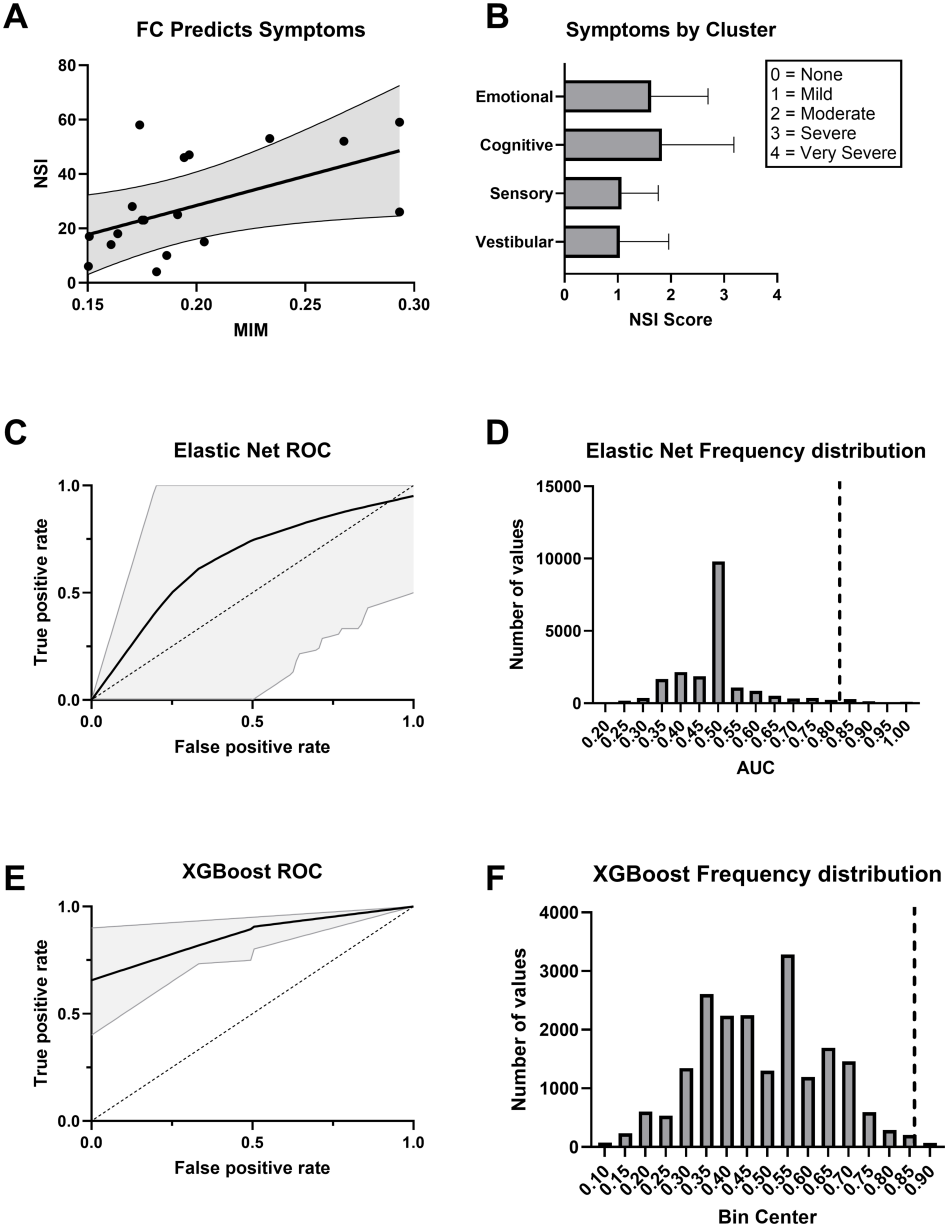
Functional connectivity explains neurobehavioral symptoms. **(A)** Higher functional connectivity (MIM) predicts greater symptom severity as measured by the Neurobehavioral Symptom Inventory (NSI). The regression line is shown with 95% confidence intervals. **(B)** Average NSI scores across symptom clusters. Scores are rated on a 0–4 scale, with 0 = None, 1 = Mild, 2 = Moderate, 3 = Severe, and 4 = Very Severe. Error bars represent ±1 SEM. **(C)** ROC curve for an Elastic Net logistic regression classifier trained on MIM. The black line indicates the mean ROC curve across 20,000 bootstraps, with 95% confidence interval shaded in gray. **(D)** Histogram displaying the frequency of AUC values under the null hypothesis for the Elastic Net model. The dashed vertical line indicates the observed AUC for the true model in each case. The observed AUC had statistically significant model performance above chance (*p* < 0.05). **(E)** ROC curve for an XGBoost classifier trained on MIM. The black line indicates the mean ROC curve across 20,000 bootstraps, with 95% confidence interval shaded in gray. **(F)** Histogram displaying the frequency of AUC values under the null hypothesis for the XGBoost model. The dashed vertical line indicates the observed AUC for the true model in each case. The observed AUC had statistically significant model performance above chance (*p* < 0.05).

MIM significantly predicted total symptom burden as measured by the Neurobehavioral Symptom Inventory (NSI) total score in a linear regression model (Figure 7A). Symptoms were distributed across emotional, cognitive, sensory, and vestibular domains (Figure 7B). MIM also effectively distinguished mTBI patients with moderate to severe symptoms (NSI total score ≥ 25) from those with mild or no symptoms (NSI total score < 25) when used as a predictor in both Elastic Net logistic regression (AUC = 0.824 [95% CI: 0.33–1]) and XGBoost classifier models (AUC = 0.865 [95% CI: 0.80–0.95]; Figures 7C,E). Permutation testing confirmed that the observed model performance was statistically significant (*p* < 0.05; Figures 7D,F).

Model performance declined when demographic covariates were added. The Elastic Net model, including MIM, age, and sex, yielded an AUC of 0.661 (95% CI: 0–1) and did not reach statistical significance (*p* = 0.125; Supplementary Figures S5A,B). A model using only age and sex performed worse than chance (AUC = 0.421, 95% CI: 0–0.583; *p* = 0.698; Supplementary Figures S6A,B). In contrast, the XGBoost model that included MIM, age, and sex maintained strong performance (AUC = 0.874, 95% CI: 0.733–1; *p* < 0.05; Supplementary Figures S5C,D), while the XGBoost model trained solely on demographics showed reduced accuracy (AUC = 0.732, 95% CI: 0.625–0.833) and was not statistically significant (*p* = 0.147; Supplementary Figures S6C,D). These results demonstrate that including MIM as a predictor significantly improved classification of symptom severity, reinforcing its potential utility as a functional biomarker in chronic mTBI.

## Discussion

### Overview and key findings

This study investigated whether RS-EEG features, including spectral power, microstate metrics, and functional connectivity, can differentiate individuals with chronic mTBI from healthy controls. Using source-reconstructed RS-EEG, we first assessed group-level differences. Subsequently, we utilized machine learning models to evaluate the capability of FC metrics to classify injury status and predict symptom severity. Although spectral power and microstate metrics did not reveal significant differences between groups, functional connectivity measured via MIM demonstrated robust and widespread hyperconnectivity in the mTBI group. These were identified up to nearly 8 years post-injury and significantly predicted symptom severity and injury status, highlighting EEG-derived connectivity as an ongoing challenge for mTBI patients that may contribute to chronic symptomology.

### Spectral power and microstate metrics lack sensitivity in chronic mTBI

No group differences in spectral power were observed, consistent with prior reports in both the subacute (<3 months) and chronic (>10 years) phases (32, 43). Although group means were unchanged, spectral measures have been linked to symptoms, including associations with memory, injury severity, and psychological problems (32, 43). Other studies, however, have identified abnormalities, such as increased theta and reduced beta power in the acute phase, altered frontal alpha/beta asymmetry persisting beyond 9 months, and reduced beta with elevated delta/gamma activity in adolescents up to 12 months post-injury (31, 44, 45). These discrepancies, together with the fact that spectral power primarily reflects local rather than network-level activity (16), suggest limited sensitivity to the distributed network disruptions typical of mTBI. Accordingly, spectral power appears to have low value for screening or longitudinal monitoring, and measures of functional connectivity may provide greater specificity and clinical relevance.

Similarly, canonical EEG microstate metrics showed no group differences. Spatial configurations and temporal parameters (duration, occurrence, coverage) were preserved in chronic mTBI. Effects have been reported when analyses were stratified by symptom severity, including shorter microstate durations and reduced alpha/theta activation in patients with moderate-to-severe neuropsychological impairments (46). However, because such effects are not evident at the group level, canonical microstates also appear limited as stand-alone biomarkers. By contrast, network-based microstates derived from MEG, defined from whole-brain connectivity rather than scalp voltage maps, have shown higher classification accuracy (47). While this approach may better capture large-scale network disruptions, MEG’s cost and limited availability constrain its clinical feasibility.

#### Persistent functional hyperconnectivity in chronic mTBI

Our MIM-based functional connectivity analysis revealed significant and widespread increases in broadband, theta, beta, and gamma bands in the mTBI group. This pattern is strikingly similar to Wu et al., who also reported increased connectivity in theta, beta, and gamma, but not alpha or delta, in professional boxers with a history of repetitive mTBI (48). The alignment across studies may reflect shared neural signatures of chronic-stage injury, as both examined participants more than a year post-injury and with likely cumulative exposure. One distinction is that Wu et al. observed hemisphere-specific effects, with stronger FC in the right hemisphere, potentially reflecting asymmetric head impacts in boxing. Our cohort’s more varied injury mechanisms may have produced a more bilateral pattern.

In contrast, studies of patients in the acute-to-subacute phase have sometimes reported reduced connectivity across multiple frequency bands (47). Such decreases are less common than early hyperconnectivity, which is often observed soon after injury and is thought to reflect compensatory recruitment of alternative pathways (16). Methodological differences may also play a role. Dynamic MEG-based analyses may be more sensitive to localized hub or long-range disruptions, whereas static EEG-based approaches capture broader global patterns. Stage of injury, analytic method, and sample characteristics likely explain why reductions are more apparent acutely, while chronic-phase data, including the present results, tend to show hyperconnectivity.

The compensatory hyperconnectivity model suggests that early increases in connectivity support recovery, but persistence into the chronic phase may signal inefficient signaling, maladaptive reorganization, or ongoing dysregulation (16). The absence of alpha and delta effects in chronic cohorts may further indicate that these bands are more sensitive to acute-stage disruptions, while later variability reflects divergent recovery trajectories and injury profiles. Source-level EEG connectivity metrics such as MIM align more closely with anatomical networks than traditional sensor-level approaches (49), reinforcing the validity of the present findings. Future multimodal work combining EEG with diffusion MRI will be critical to determine whether chronic hyperconnectivity reflects sustained compensation, unresolved structural injury, or contributes to long-term metabolic stress and neurodegenerative risk.

Several biological mechanisms may contribute to the persistent hyperconnectivity observed here. Chronic mTBI is associated with diffuse axonal injury, synaptic dysfunction, and disruptions in long-range white matter pathways, any of which can impair the efficiency of information transfer across cortical networks. Such disconnection may lead to increased functional coupling as networks recruit additional regions to maintain performance. Altered excitatory–inhibitory balance, including reduced GABAergic inhibition or chronic low-grade neuroinflammation, may also elevate baseline synchrony and increase directional connectivity. Whether these changes are compensatory or maladaptive likely depends on the stage of injury. Hyperconnectivity may initially support cognitive resilience by engaging alternative pathways, but persistent elevations years after injury may instead reflect inefficient signaling, increased metabolic demand, or reduced network specialization. The strong association with symptom burden in the present study is more consistent with a maladaptive or inefficient reorganization than a purely compensatory response.

#### MIM accurately classifies mTBI and tracks symptom burden

The multivariate interaction measure (MIM) robustly discriminated individuals with chronic mTBI from controls across multiple modeling approaches. A single-threshold decision rule (MIM > 0.174) achieved an AUC of 0.80, indicating strong sensitivity and specificity. More complex models, including penalized logistic regression and XGBoost, yielded similar performance and consistently identified elevated MIM as the most important predictor. Adding demographic variables such as age and sex provided little improvement, suggesting that MIM captures an injury-related signal independent of demographic factors. Thus, a single EEG-derived connectivity measure can accurately identify chronic mTBI, highlighting its promise as a simple and reliable screening tool.

These results are consistent with broader neuroimaging work showing that connectivity-based measures can classify TBI with high accuracy across modalities, including MEG, DTI, and resting-state fMRI (47, 50, 51). Unlike these approaches, which require high-cost imaging or high-density MEG/EEG, MIM maintained strong performance even with lower-density electrode arrays, underscoring its translational potential for clinical and field settings. Importantly, MIM also predicted individual symptom severity on the Neurobehavioral Symptom Inventory, with greater global connectivity associated with higher symptom burden. This relationship may reflect inefficient signaling or compensatory reorganization contributing to persistent cognitive and sensory difficulties. Longitudinal studies are needed to determine whether MIM changes track recovery or treatment response.

#### Clinical applications

These findings indicate that RS-EEG functional connectivity, quantified by MIM, has two potential clinical uses. First, elevated MIM provides a diagnostic signal, distinguishing individuals with chronic mTBI from controls with high accuracy even years after injury. Although the AUC values observed here are strong for research settings (AUC ≈ 0.79–0.89), they do not yet meet the performance thresholds typically required for clinical adoption, which include consistently higher accuracy (often greater than 0.85 to 0.90) and external validation. External validation refers to demonstrating that the model performs equivalently in fully independent cohorts, ideally collected at different sites or using different recording systems. Second, because connectivity strength scales with symptom burden, MIM also has utility for monitoring symptom severity over time. Although longitudinal studies are needed before applying MIM to treatment-response tracking, the current results support its use as both a diagnostic aid and a symptom-monitoring biomarker in chronic mTBI.

#### Comparison with existing biomarkers

It is important to consider how RS-EEG functional connectivity compares with currently available blood and imaging biomarkers. Blood-based markers such as GFAP and UCH-L1 primarily detect acute cellular injury, including astrocytic and axonal damage, but these signals diminish rapidly and show limited utility for identifying persistent dysfunction in the chronic stage. Structural MRI is often normal in individuals with chronic mTBI, and even advanced diffusion or functional MRI detects abnormalities in only a subset of patients. These modalities capture structural or microstructural injury but may not reflect how well cortical systems *communicate* once cellular-level processes have stabilized. In contrast, RS-EEG connectivity indexes distributed network function and reveals alterations in large-scale communication that can persist long after cellular injury has resolved. This distinction is important because chronic symptoms may arise not from ongoing cell damage but from lingering inefficiencies in network-level coordination. Thus, EEG-based functional connectivity provides a complementary biomarker that detects physiologic abnormalities often missed by blood or imaging assays, and it may be particularly valuable for characterizing chronic-stage dysfunction where existing biomarkers have limited sensitivity.

#### Interpreting EEG functional connectivity relative to MRI-based measures

FC derived from EEG and from functional MRI reflects fundamentally different physiological processes and should not be expected to show strong correspondence. EEG-based connectivity captures fast, frequency-specific interactions driven by neuronal synchronization, whereas fMRI-based connectivity reflects slow (<0.1 Hz) hemodynamic correlations that are only indirectly related to neural activity (52, 53). As a result, correspondence between EEG and fMRI connectivity is limited and varies with frequency band, brain region, analytic method, and behavioral state (54, 55). Conflicting findings in the TBI literature likely arise from these methodological differences, as well as heterogeneity in injury stage, outcome measures, and population studied. In particular, pediatric and adult TBI differ in neurodevelopmental context and compensatory capacity, limiting direct generalization across age groups. These considerations highlight the need to interpret EEG- and MRI-based connectivity as complementary, modality-specific biomarkers rather than interchangeable measures, especially for point-of-care and chronic-stage assessment.

#### Limitations

This study has several limitations. First, the relatively small sample size, particularly within the mTBI group (*n* = 18), limits statistical power and constrains the generalizability of the findings. Although the observed effects were supported by cross-validation and permutation testing, larger and more demographically diverse cohorts will be essential to confirm the robustness and reproducibility of the connectivity patterns and model performance. Second, because this was a cross-sectional study, we cannot determine the temporal stability of RS-EEG abnormalities or whether they change with recovery or treatment. Third, the absence of an independent external validation cohort prevents evaluation of model generalizability across different sites, populations, and recording systems. Finally, although we controlled for age and sex, other potential confounders (e.g., sleep, medications, comorbidities) may also influence RS-EEG measures. These limitations highlight the need for larger, longitudinal, multisite studies to validate RS-EEG functional connectivity as a clinically actionable biomarker for chronic mTBI.

#### Future directions towards scalable, non-invasive biomarkers of chronic mTBI

EEG offers advantages in accessibility, cost-efficiency, and scalability relative to imaging modalities such as fMRI or MEG, positioning it well for longitudinal and field-based applications. The present findings indicate that functional EEG measures, particularly MIM, may serve as durable indicators of persistent network disruption in chronic mTBI. Importantly, the robust effects observed even with reduced montages suggest that lower-density or portable systems could yield clinically meaningful information, supporting real-world feasibility.

Future research should prioritize replication in larger and more diverse cohorts, along with longitudinal designs that assess whether RS-EEG hyperconnectivity remains stable, resolves, or evolves over time and whether it predicts cognitive or functional outcomes. Mechanistic studies integrating EEG with complementary modalities such as fMRI, PET, or diffusion MRI will also be important for clarifying whether elevated connectivity reflects compensatory reorganization, inefficient signaling, or other chronic neurophysiological processes.

If validated in larger and independent cohorts, this approach may help address an unmet need in neurotrauma services for objective, scalable functional biomarkers suitable for deployment in major trauma centers, as highlighted in the DASIC matrix for traumatic brain injury care (56). By emphasizing non-invasive, low-burden acquisition and robustness to reduced electrode coverage, this work aligns with priorities for improving post-acute and chronic TBI assessment outside of specialized imaging facilities.

## Conclusion

Overall, our findings demonstrate that functional connectivity, quantified by MIM, is highly sensitive to chronic mTBI. This hyperconnectivity effectively distinguishes individuals with mTBI from healthy controls and robustly predicts symptom severity across multiple machine learning approaches. Resting-state EEG combined with MIM offers an accessible, accurate tool for detecting network-level disruptions in patients with chronic mTBI. With further validation, RS-EEG could be incorporated into NIH and DoD biomarker frameworks that emphasize multimodal characterization of TBI. In particular, RS-EEG aligns with the functional biomarker domain of the CBI-M initiative (7) and may complement clinical, blood-based, and imaging modifiers to improve chronic-stage classification and support more comprehensive assessment pathways for persistent brain dysfunction.

## Data Availability

The datasets presented in this study can be found in the the Federal Interagency Traumatic Brain Injury Research (FITBIR) repository, further inquiries can be directed to the corresponding author.

## Supplementary material

SUPPLEMENTARY FIGURE S1 Mean Microstate Topographical Plots by Group. Topographical representations of the mean EEG microstates **(A–E)** for the case and control groups. Each scalp map shows the average spatial distribution of electrical potentials associated with the corresponding microstate.

SUPPLEMENTARY FIGURE S2 Permutation distributions of AUC (Area Under the Curve) values for each model. Histograms display the frequency of AUC values under the null hypothesis for all three models **(A–C)**. The dashed vertical line indicates the observed AUC for the true model in each case. In all comparisons, the observed AUC had statistically significant model performance above chance (*p* < 0.05).

SUPPLEMENTARY FIGURE S3 Performance of group classifier models including Age and Sex Covariates. **(A)** ROC curve for an Elastic Net logistic regression classifier trained on MIM, age, and sex. The black line indicates the mean ROC curve across 20,000 bootstraps, with 95% confidence interval shaded in gray. **(B)** Histogram displaying the frequency of AUC values under the null hypothesis for the elastic net model. The dashed vertical line indicates the observed AUC for the true model in each case. The observed AUC had statistically significant model performance above chance (*p* < 0.05). **(C)** ROC curve for an XGBoost classifier trained on MIM, age, and sex. The black line indicates the mean ROC curve across 20,000 bootstraps, with 95% confidence interval shaded in gray. D: Histogram displaying the frequency of AUC values under the null hypothesis for the XGBoost model. The dashed vertical line indicates the observed AUC for the true model in each case. The observed AUC had statistically significant model performance above chance (*p* < 0.05).

SUPPLEMENTARY FIGURE S4 Performance of demographic group classifier models (Age and Sex Predictors only). **(A)** ROC curve for an Elastic Net logistic regression classifier trained on age and sex. The black line indicates the mean ROC curve across 20,000 bootstraps, with 95% confidence interval shaded in gray. **(B)** Histogram displaying the frequency of AUC values under the null hypothesis for the Elastic Net model. The dashed vertical line indicates the observed AUC for the true model in each case. The observed AUC had statistically significant model performance above chance (*p* < 0.05). **(C)** ROC curve for an XGBoost classifier trained on age and sex. The black line indicates the mean ROC curve across 20,000 bootstraps, with 95% confidence interval shaded in gray. **(D)** Histogram displaying the frequency of AUC values under the null hypothesis for the XGBoost model. The dashed vertical line indicates the observed AUC for the true model in each case. The observed AUC had statistically significant model performance above chance (*p* < 0.05).

SUPPLEMENTARY FIGURE S5 Performance of symptom classifier models including Age and Sex Covariates. **(A)** ROC curve for an Elastic Net logistic regression classifier trained on MIM, age, and sex. The black line indicates the mean ROC curve across 20,000 bootstraps, with 95% confidence interval shaded in gray. **(B)** Histogram displaying the frequency of AUC values under the null hypothesis for the elastic net model. The dashed vertical line indicates the observed AUC for the true model in each case. The observed model did not perform significantly above chance (*p* = 0.125). **(C)** ROC curve for an XGBoost classifier trained on MIM, age, and sex. The black line indicates the mean ROC curve across 20,000 bootstraps, with 95% confidence interval shaded in gray. **(D)** Histogram displaying the frequency of AUC values under the null hypothesis for the XGBoost model. The dashed vertical line indicates the observed AUC for the true model in each case. The observed AUC had statistically significant model performance above chance (*p* < 0.05).

SUPPLEMENTARY FIGURE S6 Performance of symptom classifier models trained on demographics. **(A)** ROC curve for an Elastic Net logistic regression classifier trained on age and sex. The black line indicates the mean ROC curve across 20,000 bootstraps, with 95% confidence interval shaded in gray. **(B)** Histogram displaying the frequency of AUC values under the null hypothesis for the Elastic Net model. The dashed vertical line indicates the observed AUC for the true model in each case. The observed model did not perform significantly above chance (*p* = 0.698). **(C)** ROC curve for an XGBoost classifier trained on age and sex. The black line indicates the mean ROC curve across 20,000 bootstraps, with 95% confidence interval shaded in gray. **(D)** Histogram displaying the frequency of AUC values under the null hypothesis for the XGBoost model. The dashed vertical line indicates the observed AUC for the true model in each case. The observed model did not perform significantly above chance (*p* = 0.147).
