# Supplementary figures and images for: Resting-state EEG and machine learning to investigate cortical connectivity as a biomarker in chronic mTBI
Source: Front Neurol. 2026 Jan 26;16:1721726. doi: 10.3389/fneur.2025.1721726 (PMC12883407; doi:10.3389/fneur.2025.1721726)

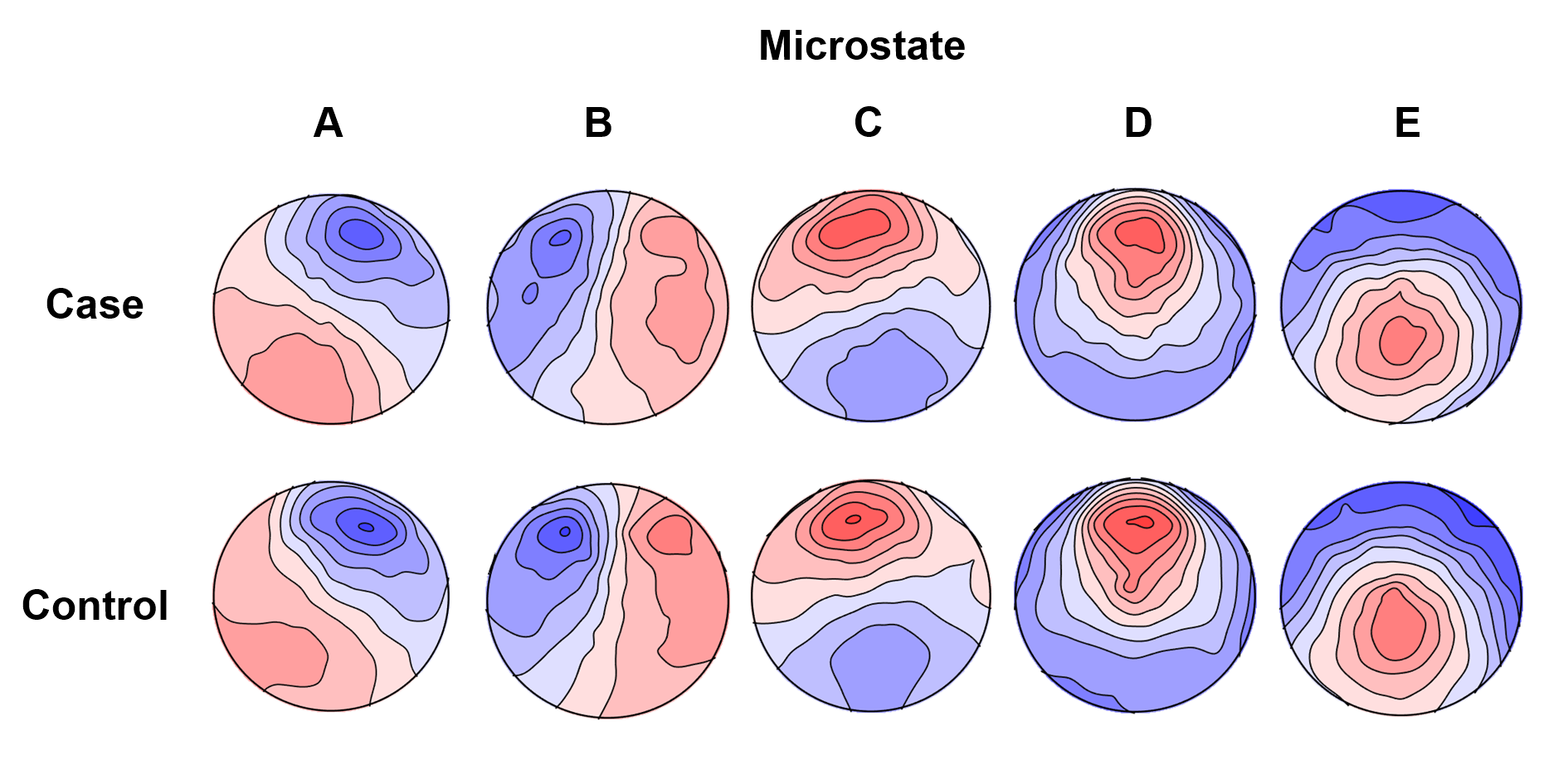

Supplement: Supplementary file 1 [file Image_1.tiff]

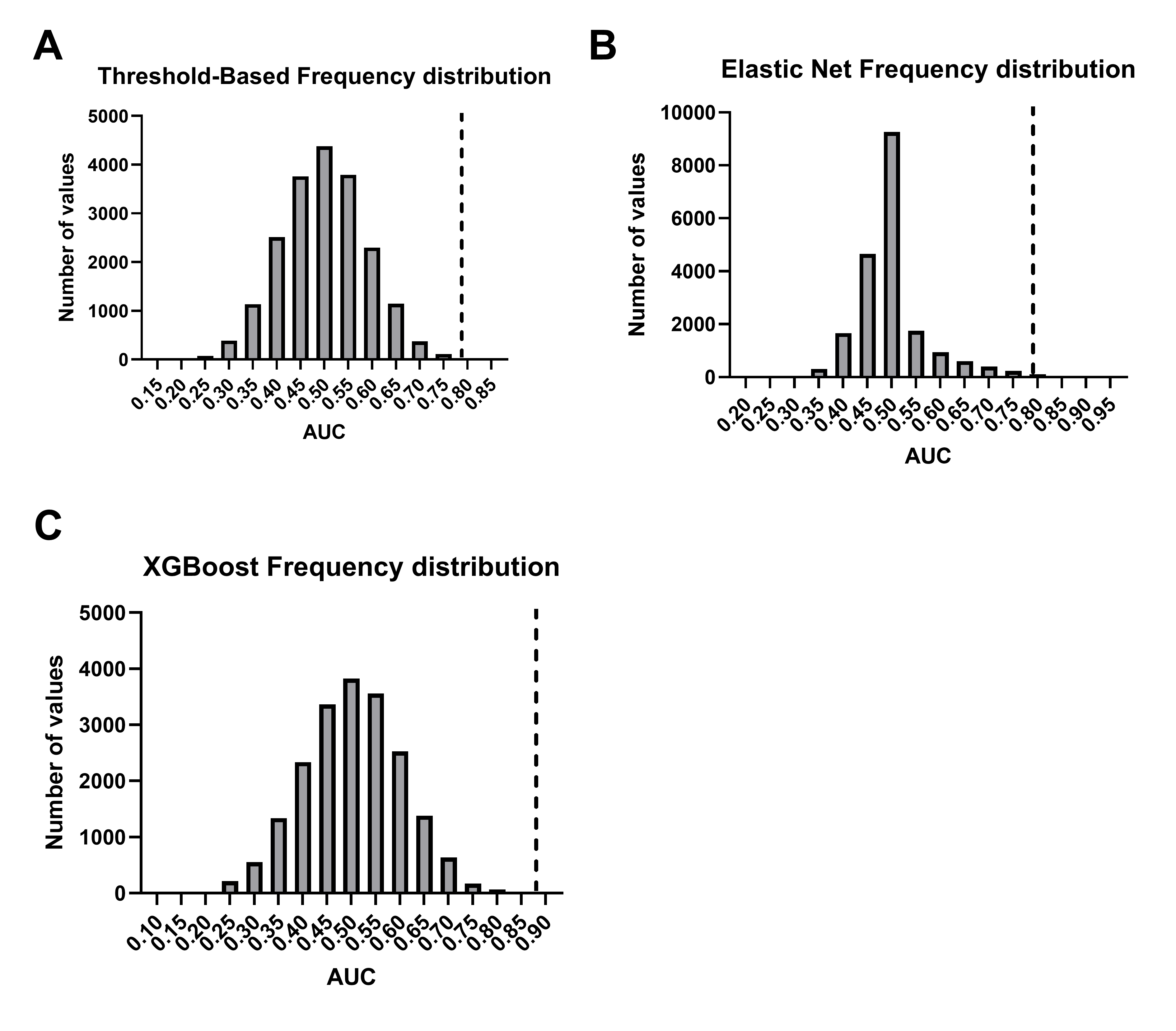

Supplement: Supplementary file 2 [file Image_2.tiff]

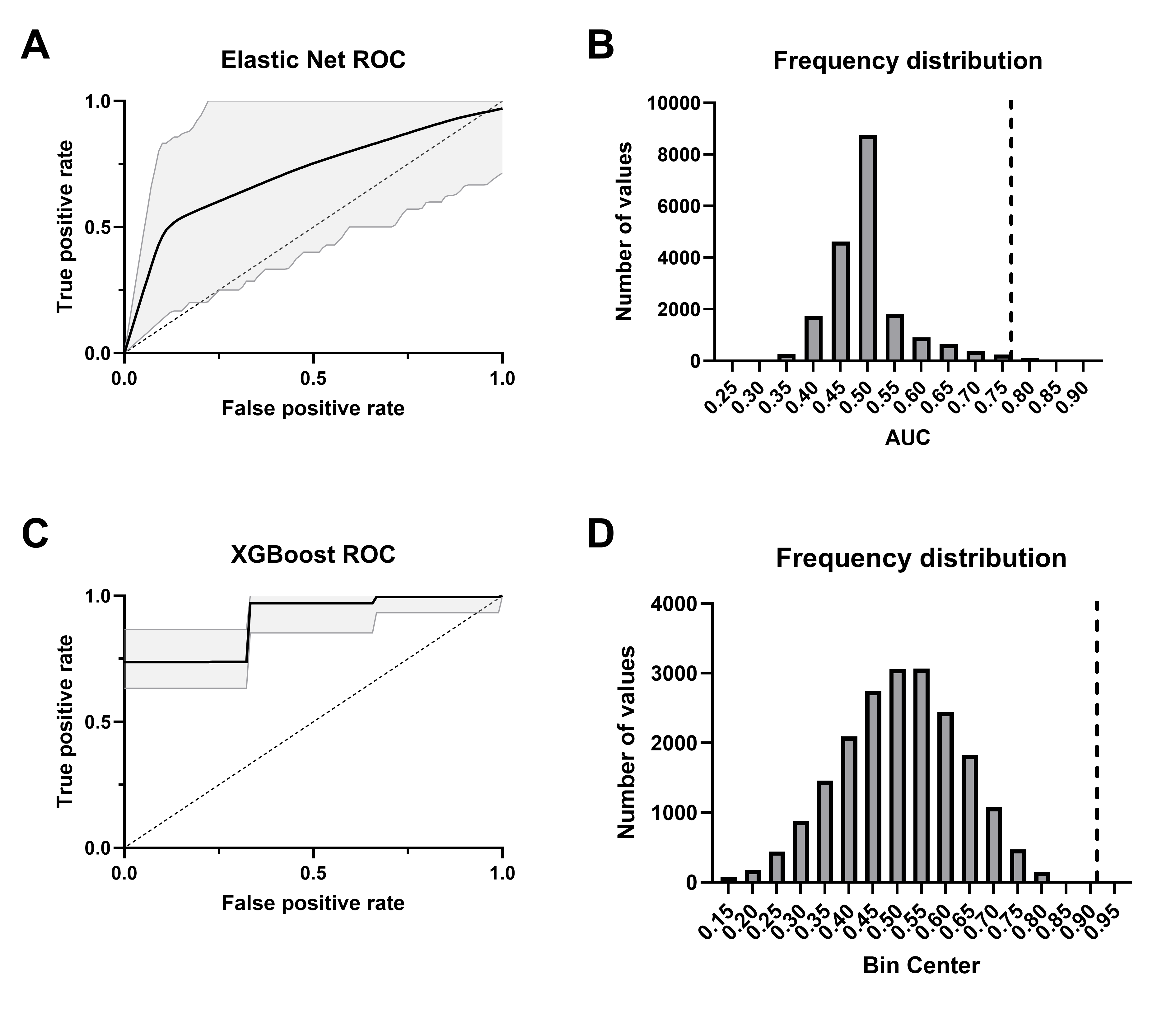

Supplement: Supplementary file 3 [file Image_3.tiff]

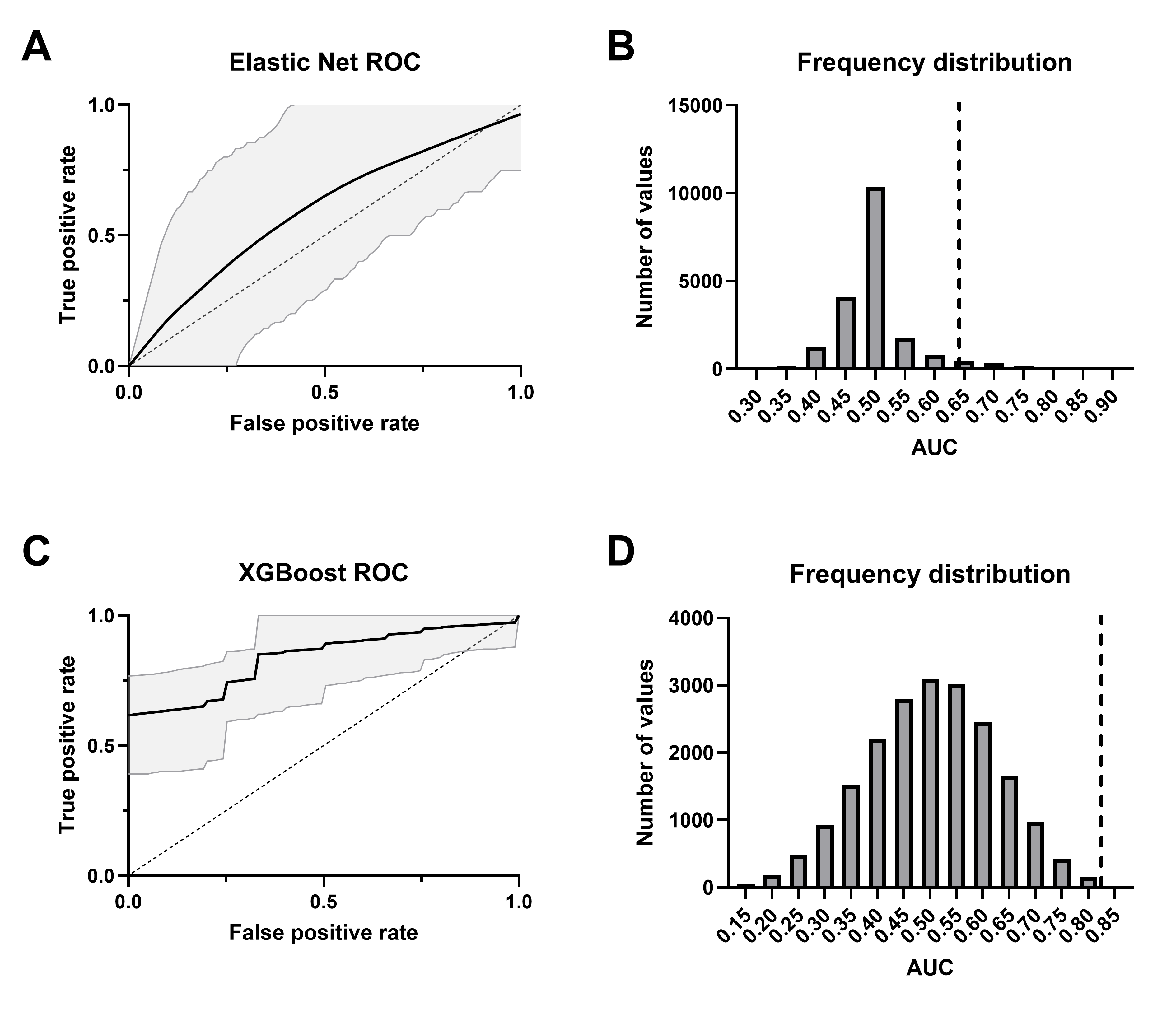

Supplement: Supplementary file 4 [file Image_4.tiff]

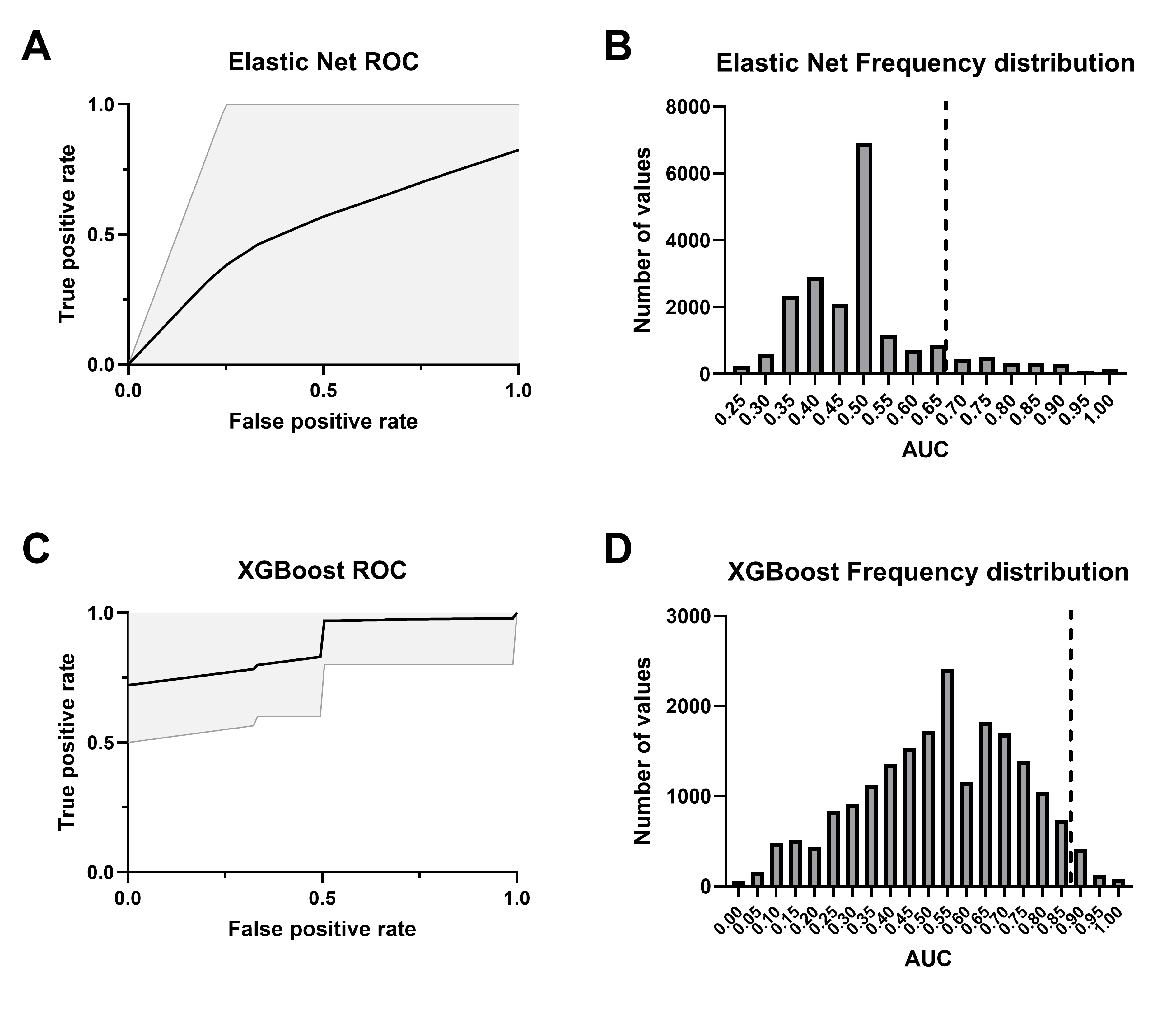

Supplement: Supplementary file 5 [file Image_5.tiff]

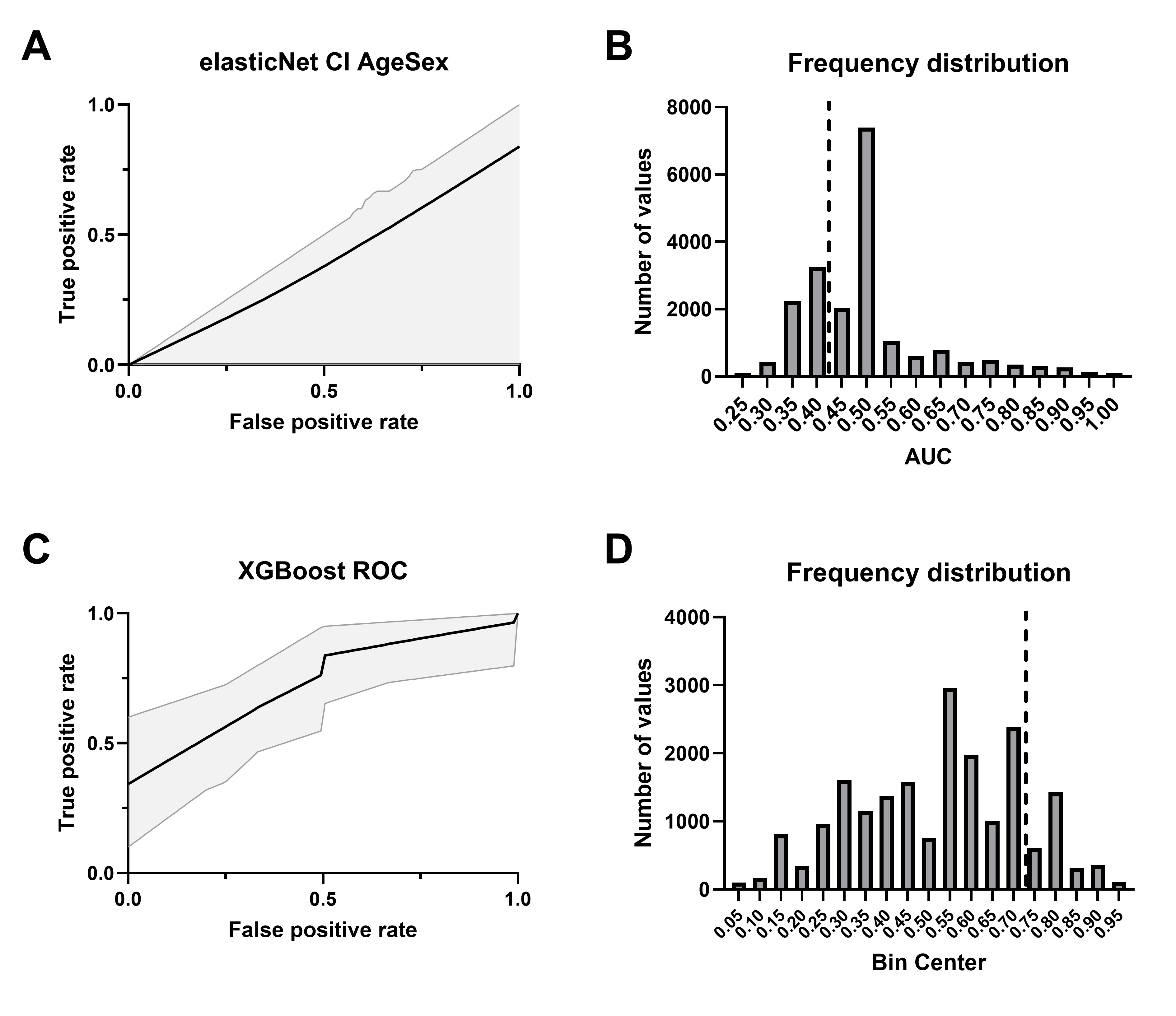

Supplement: Supplementary file 6 [file Image_6.tiff]
